# When one can infect two: a reflection on the impact of HIV discordance on child HIV infection

**DOI:** 10.4314/pamj.v5i1.56184

**Published:** 2010-05-10

**Authors:** Agnes Binagwaho, Niloo Ratnayake, Joia Mukherjee, Jules Mugabo, Etienne Karita, Elisabetta Pegurri

**Affiliations:** 1 Rwanda Ministry of Health, Kigali, Rwanda; 2 University of Virginia, Charlottesville, Virginia, United States of America; 3 Harvard Medical School, Boston, United States of America; 4 Centre of Treatment and Research on AIDS, Malaria, Tuberculosis and Others Epidemics, Kigali, Rwanda; 5 Projet San Francisco, Kigali, Rwanda; 6 UNAIDS, Kigali, Rwanda

**Keywords:** Sero-discordant couples, HIV discordance, PMTCT, children, HIV, Rwanda

## Abstract

This is an opinion piece based on data and experience from Rwanda. The authors believe this opinion piece may help improve current programs on prevention of HIV transmission from mother to child in Africa taking into account the prevalence of HIV sero-discordance in couples. The authors recommend that if we want to ensure newborns stay HIV negative, PMTCT protocols should offer a series of HIV tests linked with antenatal visits and the lactation period as well as HIV testing of current sexual partners. Moreover, if the male partner is found to be positive and the woman is negative, programs should provide intensive counseling on the use of condoms. The lives of three individuals have the potential to be changed from HIV testing and counseling. Morally, this cannot be ignored.

## Opinion

A number of studies have looked at the prevalence of HIV discordant couples in Africa [1, 4]. An article recently published in the Lancet estimated that up to 94% of new heterosexually acquired HIV infections occur within marital or cohabiting relationships in urban areas of Rwanda and Zambia. [1]. In Kigali, the capital city of Rwanda, it is estimated that up to 12% of cohabiting couples seeking voluntary counseling and testing services are HIV discordant. The proportion of discordant couples with an HIV positive man is almost the same as that of discordant couples with an HIV positive woman [1].

There is a need to better analyze the impact of HIV sero-discordance in the context of pregnancy and prevention of mother to child transmission (PMTCT). In general, PMTCT programs in the African context tend to offer one HIV testing and counseling session only to women and little follow-up is planned for women who test negative to HIV during pregnancy.

In Rwanda, where HIV prevalence among pregnant women was 4.3% in 2007, about 73% of pregnant women attending antenatal clinics agreed to undergo HIV testing and 84% had partners who agreed to undergo testing in 2009 [5]. This means that more than 27% of pregnant Rwandan women still do not know their HIV status. Also, although the great efforts to encourage the partners of pregnant women to be tested for HIV through couple counselling and testing, among women who do know their serostatus, 16% do not know the father of the child’s status. Both of these issues place the child of an HIV negative mother at risk.

The risk of HIV acquisition by pregnant women in very high prevalence settings may reach up to 10.7/100 woman years as recently shown in a study conducted in South Africa [6]. We also know that primary infection of women around pregnancy or lactation is more likely to result in HIV transmission to the child due to high viral load [7, 10].

This reality suggests the need for specific guidelines regarding HIV testing of pregnant women and their partners, with repeat testing of HIV negative pregnant women, particularly in an HIV discordant relationship.  Education about risk of acquiring new infection and the distribution of condoms should also occur at each antenatal, post natal, newborn and child visit.

In Rwanda nearly all pregnant women come at least for one antenatal care (ANC) visit. The percentage of pregnant women attending ANC that were tested for HIV increased from 10.5% in 2002 to more than 70% in 2009 (about 295,000 pregnant women) [5]. The high acceptance rate of the first test makes us expect a similarly high acceptance of a second test among HIV negative pregnant women. Special emphasis on counseling may go for those women who did not accept HIV testing on the first place.

A study on the adequacy and feasibility of a second HIV test during pregnancy at a reference Hospital in Kigali, Rwanda confirmed that a second test should be recommended for HIV negative women. In fact, among 1,342 HIV negative pregnant women at first test, 3 cases of HIV seroconversion were found (107 days on average between the first and second test for the three cases) for a cumulative incidence over 9 months of 2.1‰. The partners of two of the three women tested negative at the first test and one was of unknown HIV status. Overall, 3,28% of partners of the HIV negative pregnant women tested HIV positive at first test and 13.34% of HIV negative women did not know the HIV status of their partners [11].

A cost-effectiveness study of HIV rescreening during late pregnancy to prevent peri-natal transmission in South Africa, a country with an overall national HIV prevalence among antenatal women aged 15-49 years of 29.3% in 2008 [12], found that rescreening would prevent additional infant infections and result in net savings [13]. The minimum time interval between the initial and repeat screens would be from 3 to 18 weeks, depending on prophylactic and treatment regimens, in order for HIV rescreening to be cost saving. Overall, HIV rescreening late in pregnancy in very high-prevalence, resource-limited settings such as South Africa would be a cost-effective strategy for reducing mother-to-child transmission [13].

Each country should define its protocol according to knowledge of HIV prevalence combined with the risk of vertical infection during pregnancy and lactation. Moreover, if the male partner is found to be positive and the woman is negative, intensive counseling on the use of condoms and the need for repeat testing throughout the gestational period and during lactation should be done. The Rwanda model of couple HIV counseling and testing which promotes male involvement and ensure HIV disclosure provides a positive support environment that facilitates the management of sero-discordant results, particularly during pregnancy. HIV testing becomes and entry point for combination prevention of heterosexual transmission during pregnancy including systematic condom use, antiretroviral therapy if eligible for HIV positive male partners [14].

The lives of women but also their children have the potential to be saved from repeated HIV testing and counseling in the context of PMTCT. Morally, this cannot be ignored.
